# *Caenorhabditis elegans* models of alternating hemiplegia of childhood have dominant neuromuscular junction defects

**DOI:** 10.1242/dmm.052809

**Published:** 2026-06-02

**Authors:** Diana A. Wall, Adam M. Friedberg, Jeremy Lins, Roza Khalifa, Sienna Partipillo, Anne C. Hart

**Affiliations:** ^1^ Brown University Department of Molecular Biology, Cell Biology, and Biochemistry, Providence, RI 02912, USA; ^2^ Robert J. and Nancy D. Carney Institute of Brain Science, Brown University, Providence, RI 02912, USA; ^3^ Brown University Department of Neuroscience, Providence, RI 02912, USA

**Keywords:** ATP1A3, EAT-6, Na^+^/K^+^ ATPase, Alternating hemiplegia of childhood, NCX-4, Aldicarb, Levamisole

## Abstract

Dominant missense mutations in *ATP1A3*, encoding ATPase Na^+^/K^+^-transporting subunit alpha3, can cause the neurological disorder alternating hemiplegia of childhood (AHC), but how these mutations lead to AHC remains unclear. Here, we established the first *Caenorhabditis elegans* AHC models by introducing AHC-causing ATP1A3 mutations (i.e. D801N, E815K, L839P and G947R substitutions) into the orthologous gene *eat-6* by using CRISPR/Cas9. Homozygous *C. elegans* AHC model animals have recessive developmental defects. Heterozygous AHC model animals have dominant defects in neuromuscular junction (NMJ) function that are inconsistent with haploinsufficiency and dominant sleep or arousal defects. Previous work in a *Drosophila* G755S AHC model found that loss of a K^+^-dependent, Na^+^/Ca²^+^ exchanger exacerbated neuronal defects. We introduced a loss-of-function allele of the orthologous *C. elegans ncx-4* gene into *C. elegans* AHC models. Loss of *ncx-4* function mutation did not consistently alter *C. elegans* AHC model defects across alleles. Our results established novel *C. elegans* models of AHC with robust phenotypes, demonstrating that AHC-causing ATP1A3 variants disrupt NMJ function, and providing proof-of-concept for discovering cross-species modifiers of AHC-related phenotypes.

## Teaser

**Summary:** We report the first *C. elegans* models of alternating hemiplegia of childhood. D801N, E815K, L839P and G947R AHC model animals have recessive development defects and dominant neuromuscular defects.

## INTRODUCTION

Mutations in ATPase Na^+^/K^+^-transporting subunit alpha3 (ATP1A3) cause a spectrum of disorders with overlapping neurological and developmental symptoms (Vezyroglou et al., 2022). Beyond symptomatic treatments, there are no effective therapies for ATP1A3-related diseases, placing a heavy burden on thousands of patients and families (Auvin, 2024; Neville and Ninan, 2007; Samanta, 2020). We focus on dominant *de novo* missense mutations in ATP1A3 that cause alternating hemiplegia of childhood (AHC). AHC is typically defined by episodes of paralysis (plegia) that affect one or both sides of the body. Onset occurs in infancy, and patients often present with additional symptoms, such as abnormal eye movements, dystonia, seizures, developmental delays, intellectual disabilities and progressive brain atrophy (Vezyroglou et al., 2022; Heinzen et al., 2012; Rosewich et al., 2012). While over 40 different AHC-associated ATP1A3 variants have been identified, the most frequently occurring *ATP1A3* alleles causing AHC are amino acid (aa) substitutions D801N, E815K and G947R (Viollet et al., 2015; Heinzen et al., 2012; Verret and Steele, 1971; Vezyroglou et al., 2022; Rosewich et al., 2012; Yang et al., 2014; Li et al., 2022). Each of these three AHC alleles is associated with slightly different symptoms. For example, E815K/+ patients are more likely to develop epilepsy, while cardiac muscle function defects are most common in D801N/+ patients (Moya-Mendez et al., 2021, 2025; Funk et al., 2025; Panagiotakaki et al., 2015; Viollet et al., 2015; Yang et al., 2014).

ATP1A3 is a catalytic subunit of the P-type Na^+^/K^+^-ATPase that exports Na^+^ and imports K^+^ ions; maintaining appropriate electrochemical Na^+^ and K^+^ gradients is critical for establishing physiologic membrane potential and ionic balance (Kaplan, 2002). ATP1A3 is one of four human Na^+^/K^+^-ATPase alpha subunits that function as heterodimers with ATP1B family beta subunits. Beta subunits are required for maturation, transport to the membrane and cation pump function of ATP1A proteins (Geering, 2001; Kaplan, 2002). The impact of mutations on ATP1A3 levels, localization and function in AHC patients has been carefully examined. *ATP1A3* mutations in AHC patients do not alter *ATP1A3* mRNA levels, and most AHC mutations (including D801N and E815K substitutions) do not dramatically change ATP1A3 protein levels (Heinzen et al., 2012; Weigand et al., 2014; Blanco-Arias et al., 2009). While AHC-causing *ATP1A3* alleles do decrease ATP1A3 function, multiple lines of evidence suggest that AHC pathophysiology is more than a simple loss of ATP1A3 pump activity (Simmons et al., 2018; Sweadner et al., 2019). Other cellular and molecular processes are likely impaired by AHC mutations, leading to dysfunction and patient symptoms.

The *Caenorhabditis elegans* ortholog for all four human ATP1A family proteins is EAT-6 (Davis et al., 1995), which is 72% identical in aa sequence to ATP1A3. Complete loss of *eat-6* function is embryonic or early larval lethal, and partial loss-of-function alleles cause recessive defects in pharyngeal pumping, neuromuscular junction (NMJ) function, fecundity and mechanosensory response (Avery, 1993; Davis et al., 1995; Johnson et al., 2020; Doi and Iwasaki, 2008; Govorunova et al., 2010; Hawkins et al., 2015). *C. elegans* NMJ function can be readily examined with classic drug-challenge assays using the acetylcholinesterase inhibitor aldicarb or the nicotinic agonist levamisole (Rand, 2007b; Lewis et al., 1980b; Mahoney et al., 2006; Hobson et al., 2017). Partial loss of *eat-6* function causes recessive hypersensitivity to aldicarb, and some alleles also cause recessive hypersensitivity to levamisole (Govorunova et al., 2010; Doi and Iwasaki, 2008). Therefore, decreased EAT-6 function disrupts NMJ function, and some alleles cause post-synaptic defects. EAT-6 function in motor neurons is critical for normal NMJ function (Doi and Iwasaki, 2008). For some alleles, EAT-6 function in muscles is critical for normal levamisole response and proper trafficking of post-synaptic nicotinic acetylcholine receptors (Doi and Iwasaki, 2008). The deeply conserved sequence and function of mammalian and *C. elegans* ATP1A family proteins support the use of invertebrate models for studying AHC-causing ATP1A3 mutations.

Here, we report the creation of *C. elegans* AHC models for the most-severe and recurrent AHC patient alleles D801N, E815K and G947R, as well as the less-severe AHC allele L839P. These AHC model animals have recessive developmental defects, and dominant arousal and NMJ defects. Finally, we used these novel AHC models to test the cross-species relevance of the conserved K^+^-dependent, Na^+^/Ca^+^ exchanger solute carrier family 24 member 1 (SLC24A1, also known and hereafter referred to as NCKX), for which loss of function has been reported to modify defects in a *Drosophila* AHC model (Talsma et al., 2014).

## RESULTS

### Insertion of AHC-causing *ATP1A3* mutations into *C. elegans eat-6*

**Fig. 1.**
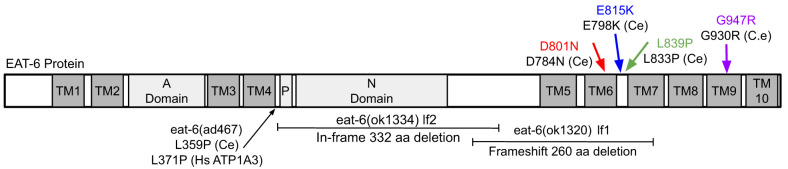
**AHC patient mutations and loss-of-function alleles in *C. elegans* EAT-6.** *C. elegans* EAT-6 contains ten transmembrane (TM) domains, one actuator (A) domain, one phosphorylation (P) site and one nucleotide-binding (N) domain. The homozygous viable, missense allele *eat-6(ad467)* has been described previously (Avery, 1993). *eat-6(ok1320)* and *eat-6(ok1334)* are homozygous lethal deletion alleles. In this study, *ok1320* is referred to as *lf1* and *ok1334* is referred to as *lf2. C. elegans* AHC models for D801N, E815K, L839P and G947R AHC patient mutations in ATP1A3 are generated in this study by editing endogenous *eat-6*. ‘*Ce*’ indicates amino acid numbering in *C. elegans*; ‘*Hs*’ indicates amino acid numbering in *Homo sapiens*.

We selected the dominant AHC-causing ATP1A3 variants D801N, E815K, L839P and G947R to be used for CRISPR/Cas9-based introduction into endogenous *C. elegans eat-6*. Whereas D801N, E815K and G947R are the most-severe and most frequently recurring *ATP1A3* alleles in AHC patients, L839P is a less-severe allele, which allows for comparison of allele severity between the *C. elegans* models. Each edit to *C. elegans eat-6* was introduced by using a single-stranded CRISPR repair template designed to include one *ATP1A3* missense mutation (i.e. D801N, E815K, L839P and G947R), as well as silent mutations to remove protospacer adjacent motif (PAM) sequences and create restriction digest sites for PCR genotyping. CRISPR control strains were generated for each *ATP1A3* allele (i.e. D801D, E815E, L839L and G947R) to control for the presence of these silent mutations (Fig. 1; Figs S2, S3). We generated two strains for each *ATP1A3* allele and for each CRISPR control (see Materials and Methods). Results from the arbitrarily selected ‘primary’ AHC model (i.e. D801N, E815K, L839P and G947R) and CRISPR control strains were very similar to results from the ‘replicate’ AHC model (i.e. D801N*, E815K*, L839P* and G947R*) and CRISPR control strains, with minor exceptions described below. Replicate strains are labeled with an asterisk (*). For clarity, we describe the *C. elegans* AHC model strains herein by using human aa numbering; formal *C. elegans* allele designations can be found in Dataset 1, ‘Strain list’.

### Developmental defects in homozygous AHC model animals

**Fig. 2.**
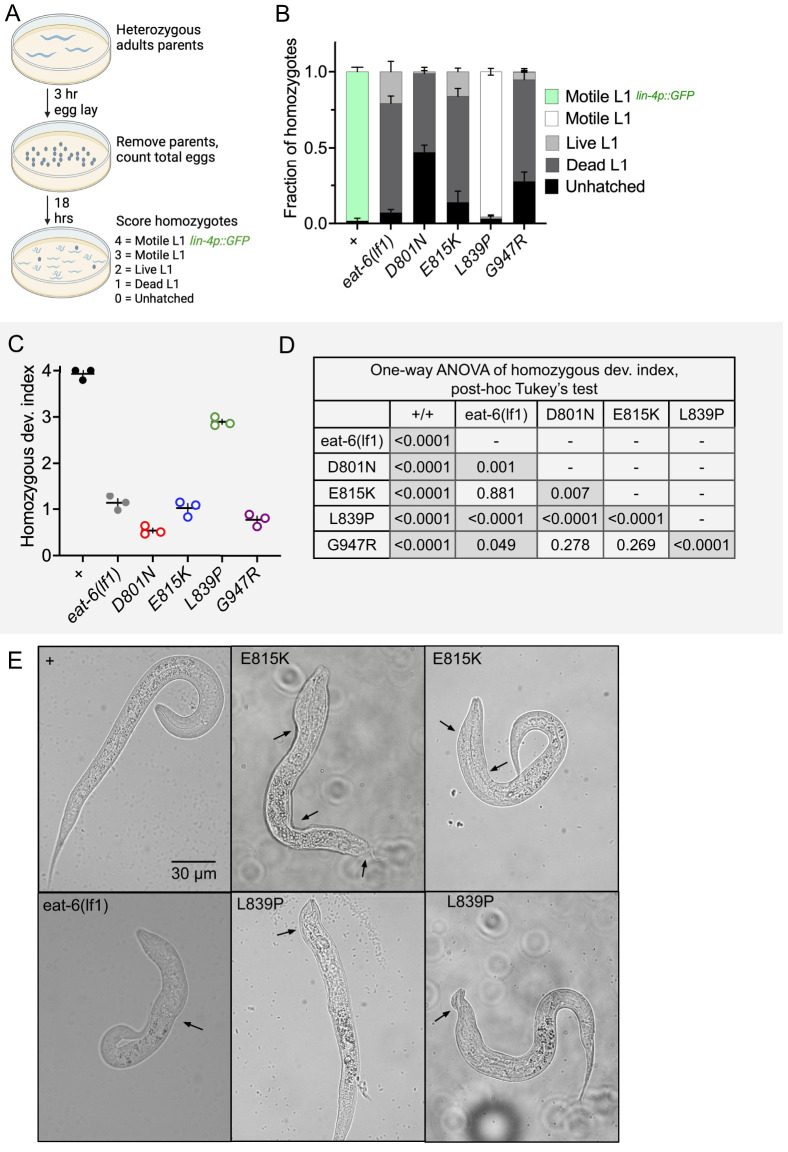
**Developmental defects of homozygous AHC model animals.** (A) Homozygous development assay and development index used in B and C, respectively. (B) Fraction of homozygous animals 18 h after egg-lay from balanced, heterozygous parents categorized as unhatched, dead L1, live L1, motile L1, motile L1 expressing *lin-4p::GFP (maIs134)*. *n*=117−211 homozygous animals per genotype scored from three biological replicates. Error bars indicate mainstem. (C) Development index score of homozygous animals presented in B. AHC model homozygotes have severe development defects. (D) One-way ANOVA of data from C with Tukey's multiple comparisons test between all pairs. Comparisons with *P*<0.05 are indicated in gray. (E) Representative images of homozygous wild-type, E815K, L839P and *eat-6(lf1)* animals 18 h after egg lay. Arrows indicate examples of abnormal body thickness. *eat-6(lf1)* is referred to as *ok1320*.

Homozygous AHC model animals had profound developmental defects. These animals either failed to hatch or failed to develop normally (Fig. 2), which was not unexpected as homozygous AHC model animals of other species also have severe homozygous phenotypes (Uchitel et al., 2021; Ng et al., 2021; Hunanyan et al., 2015). Therefore, AHC model animals were maintained as balanced heterozygotes. Heterozygous AHC model animals had one chromosome with the AHC patient mutation in *eat-6* and one balancer chromosome (*tmC12*) with an unedited functional copy of *eat-6* (e.g. D801N/+)*.* Homozygous CRISPR control and heterozygous AHC model animals were healthy, and developed into overtly normal and fertile adults without obvious locomotor defects.

Homozygous AHC model animals were produced by balanced heterozygous parents at the expected Mendelian ratio of 1:3, but homozygous AHC model animals were not viable and never developed past the first (L1) larval stage. These L1 animals were smaller than wild-type animals but always had an overtly normal body plan with a pharynx and intestine. Sometimes, homozygous AHC model animals showed abnormal body thinning, bumps or head swelling (Fig. 2E, black arrows). These phenotypes were highly variable and most frequently observed in homozygous E815K, L839P and *eat-6* loss-of-function animals.

To carefully examine the developmental defects of homozygous AHC model animals, we recorded the developmental state of homozygous AHC model progeny 18 h after an egg-lay by heterozygous parents (Fig. 2A-D). Homozygotes were categorized as (0) unhatched, (1) dead L1, (2) live L1, (3) motile L1 (that do not reach mid-L1 stage) and (4) motile L1 (that do reach mid-L1 stage) (Fig. 2A,B). Each category was assigned a developmental index score between 0 and 4, respectively, to allow comparison across lines (Fig. 2A,C,D). To determine whether an animal reached the mid-L1 stage, we examined the expression of a previously described developmental marker, *lin-4::GFP* (Feinbaum and Ambros, 1999; Olsen and Ambros, 1999; Ow et al., 2008).

Virtually all homozygous wild-type and CRISPR control animals hatched, showed coordinated locomotion and developed beyond the L1 stage. The *eat-6(ok1320)* loss-of-function allele (referred to as *lf1*) is a 260-aa frameshift deletion lacking transmembrane domains 5-7 (Fig. 1). Most homozygous *eat-6(lf1)*, E815K and G947R animals hatched, but the majority of their L1 larvae were dead. A small portion of homozygous *eat-6(lf1)*, E815K and G947R larvae were alive, with some twitching spontaneously and some only responding when prodded. However, these animals never expressed *lin-4p::GFP* and, therefore, never developed to mid-L1 stage. Homozygous D801N model animals had the most-severe development defects; almost half of the animals were unhatched and almost all L1 larvae were dead (Fig. 2). Developmental defects of homozygous D801N and G947R animals were more severe than of homozygous *eat-6(lf1)* animals, while defects of homozygous E815K animals could not be distinguished from those of homozygous *eat-6(lf1)* animals in this analysis.

Developmental defects observed in homozygous L839P model *C. elegans* were less severe. Almost all of these animals hatched and were motile, showing slow, spontaneous, sinusoidal movement (Fig. 2). Homozygous L839P model animals lived for >10 days, becoming increasingly sessile. We confirmed this for the replicate L839P* homozygous animals (*n*=30, 100% animals surviving >10 days). By day 14, about half of the homozygous L839P animals had died. Despite their prolonged survival, homozygous L839P animals failed to increase in size and never expressed *lin-4p::GFP*, which indicates a failure to develop past the mid-L1 developmental stage*.* This survival without growth or maturation is similar to that of wild-type animals hatched in the absence of food (Johnson et al., 1984). Combined, these results demonstrate that homozygous *C. elegans* AHC model animals have severe developmental defects, which prevent their growth beyond the earliest stages of development.

### AHC model animals do not have defects in dominant pharyngeal pumping, egg-laying or stress-response

Typical *eat-6* alleles, such as *eat-6(ad467)*, were identified in a forward genetic screen based on recessive pharyngeal pumping defects (Avery, 1993). As filter-feeding animals, *C. elegans* rapidly and rhythmically contract pharyngeal muscles to pump bacteria into the intestine (Avery and Horvitz, 1989; Albertson and Thomson, 1976); defective neuromuscular function can, therefore, decrease pharyngeal pumping rates (Avery, 1993; Davis et al., 1995). We examined heterozygous AHC model animals to determine if they also display pharyngeal pumping defects. Overall, the *C. elegans* AHC model animals did not consistently show altered pumping rates (Fig. S3A). We note that day-1 adult animals from the D801D*/+ CRISPR control strain had a slightly decreased pumping rate, which means D801N*/+ day-1 adults appear to have an increased pumping rate in comparison. But this result was not replicated in D801D/+ and D801N/+ animals, the pumping rates of which are almost identical (Fig. S3A). We also assessed pharyngeal pumping of day-8 adult animals but no consistent pharyngeal pumping defects were observed. We note that E815E*/+ animals had increased pharyngeal pumping rate, when compared to wild-type control animals (Fig. S3B); this was not seen in E815E/+ animals. Both primary and replicate strains of L839L/+ and L839P/+ showed normal pharyngeal pumping rates. Overall, we conclude pharyngeal pumping is not strongly or consistently altered by dominant AHC mutations in *eat-6.*

The standard food source for *C. elegans* is the *E. coli* strain OP50, but rearing *C. elegans* on different *E. coli* strains can reveal subtle feeding defects. The DA837 *E. coli* strain grows in large, sticky clumps and is more difficult for *C. elegans* to ingest (Avery and Shtonda, 2003). In contrast, *E. coli* strain HB101 has lower viscosity and is easier for feeding-defective animals to consume (Davis et al., 1995). Heterozygous AHC model animals did not show overt growth-rate or fertility changes when grown on DA837 or HB101 *E. coli* strains. Furthermore, allowing homozygous AHC model progeny to hatch on HB101 *E. coli* did not alter larval development. Appearance and locomotion of heterozygous AHC model *C. elegans* on all three bacterial strains was indistinguishable from wild-type or CRISPR control strains. We, therefore, conclude that failure to develop into homozygous AHC model animals was probably not due to feeding defects.

Typical *eat-6* allele animals also have recessive egg-laying defects (Davis et al., 1995), as coordinated vulval muscle contractions are required to lay eggs (White et al., 1986). Heterozygous AHC model animals did not show dominant defects in egg-laying (Fig. S3C).

Temperature stress is a common trigger of hemiplegic and dystonic episodes in AHC patients (Heinzen et al., 2014; Salles et al., 2021; Brashear et al., 2008), and AHC mouse models have temperature-induced paroxysmal episodes (Uchitel et al., 2021; Helseth et al., 2018; Terrey et al., 2025; Isaksen et al., 2017). We exposed heterozygous AHC model *C. elegans* to a variety of acute or chronic heat- or cold-stress to determine whether these can induce dramatic survival or locomotion defects. After a 17-h 4°C or a 4-h 0°C acute cold shock, no survival or locomotion defects were observed in heterozygous D801N/+, E815K/+ or L839P/+ model animals (*n*=60 animals of three biological replicates for each cold shock paradigm; survival rate: 100%). After a 2.5-h acute heat shock at 35°C, heterozygous AHC model animals showed an insignificantly increased survival rate compared with that of their CRISPR control strains (Fig. S3D). Last, long-term growth at 12°C or 27°C did not overtly alter survival or locomotion defects in AHC model animals. In combination, these studies suggest that heterozygous AHC model *C. elegans* cannot be distinguished from control animals based on obvious behavioral defects.

### Dominant NMJ defects in AHC model animals: aldicarb hypersensitivity

Animals carrying homozygous viable *eat-6* partial loss-of-function mutations show normal locomotion but drug challenge assays have revealed recessive NMJ defects (Doi and Iwasaki, 2008; Hawkins et al., 2015; Sorkaç et al., 2016; Johnson et al., 2020; Govorunova et al., 2010). *C. elegans* NMJ function is frequently examined by using the acetylcholinesterase inhibitor aldicarb, an. In the presence of aldicarb, acetylcholine accumulates at the NMJ and irreversibly induces muscle contraction. Even in wild-type *C. elegans*, exposure to aldicarb will lead to immobilization, due to constant muscle contraction, over time or with increasing dose. In almost every case, an altered rate of immobilization on aldicarb broadly indicates defective NMJ function or developmental defects at the NMJ. Note that aldicarb-induced immobilization is not directly equivalent to dystonia or paralysis observed in AHC patients but does serve as a robust assessment of overall NMJ function. The aldicarb assay alone cannot determine whether NMJ dysfunction results from defects in neurotransmitter (acetylcholine and/or GABA) release and/or post-synaptic response by muscles (Rand, 2007a; Mahoney et al., 2006). We used two different formats of aldicarb assay to examine NMJ function in heterozygous *C. elegans* AHC model animals, i.e. dose–response and time–response assay format. We found that *C. elegans* AHC model animals had dominant NMJ defects in both assay formats.

**Fig. 3.**
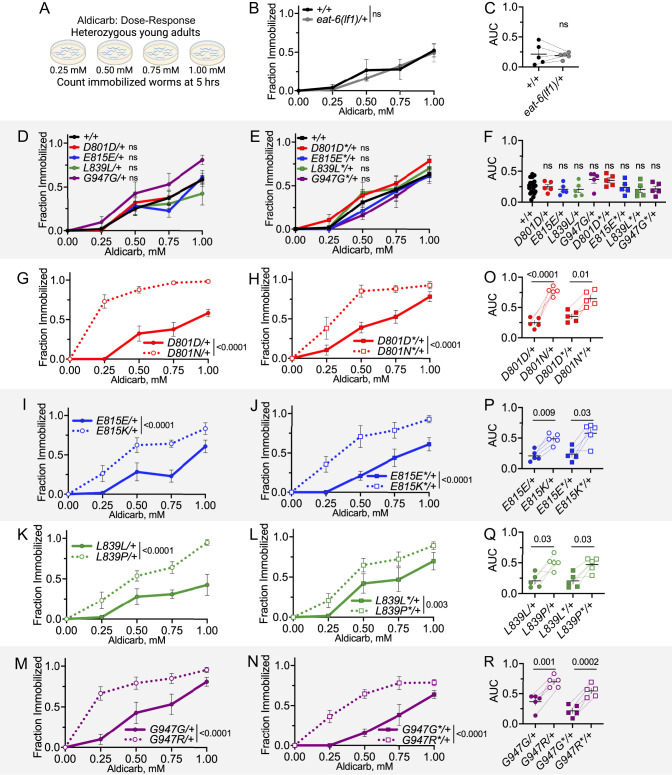
**Dominant NMJ defects of AHC model animals: aldicarb dose–response assay.** (A) Schematic of aldicarb dose–response assay. Wild-type (+/+) and heterozygous *eat-6(lf1)/+* young-adult animals were exposed to 0, 0.25, 0.5, 0.75 or 1 mM aldicarb for 5 h, and numbers of immobilized animals were recorded. (B) Dose–response curve for *eat-6(lf1)/+* animals; *lf1* is referred to as *ok1320*. Two-way ANOVA. (C) Area under the curve (AUC) from each trial described in B. Paired *t*-test. (D,E) Dose–response curves for heterozygous CRISPR control primary and replicate strains. Accumulated +/+ data were plotted for visual purposes, but two-way ANOVA was tested for CRISPR control and +/+ animals run in parallel. (F) AUC from each trial in D and E. Paired *t*-test between each CRISPR control and +/+ animals run in parallel. (G-N) Dose-response curves for heterozygous primary and replicate CRISPR control, and AHC model strains. CRISPR control data are the same as those shown in D and E. Two-way ANOVA between each CRISPR control and AHC model strain. (O-R) AUC for each trial in G-N. Paired *t*-test between each CRISPR control and its corresponding AHC model strain tested in parallel. For all two-way ANOVA analysis, the displayed *P*-value results from genotype as the source of variation. ns, not significant (*P*>0.05). All animals in B-R have one copy of the *tmC12* balancer represented by ‘+’. For each genotype five biological replicates were tested. For each trial, *n*=10-15 animals per genotype per dose. Error bars indicate mean±s.e.m.

In the dose–response aldicarb assay, we measured the fraction of animals immobilized after 5 h on 0, 0.25, 0.50, 0.75 or 1 mM aldicarb (Fig. 3A). In this assay, heterozygous *eat-6(lf1)/+* animals responded normally to aldicarb [based on both two-way ANOVA versus balanced wild-type animals, and on area under the curve (AUC) values using a paired *t*-test, Fig. 3B,C]. Primary and replicate CRISPR control strains also responded normally to aldicarb when compared to balanced wild-type animals (Fig. 3D-F). All eight primary and replicate heterozygous AHC model strains showed dominant hypersensitivity to aldicarb when compared to their heterozygous CRISPR control strains (Fig. 3D-R). The significant aldicarb hypersensitivity of all AHC model strains was seen in both two-way ANOVA analysis of the dose–response curves and in paired *t*-test analysis of AUC values. This indicates all four *C. elegans* AHC models show dominant defects in NMJ function, while *eat-6(lf1)/+* animals do not.

**Fig. 4.**
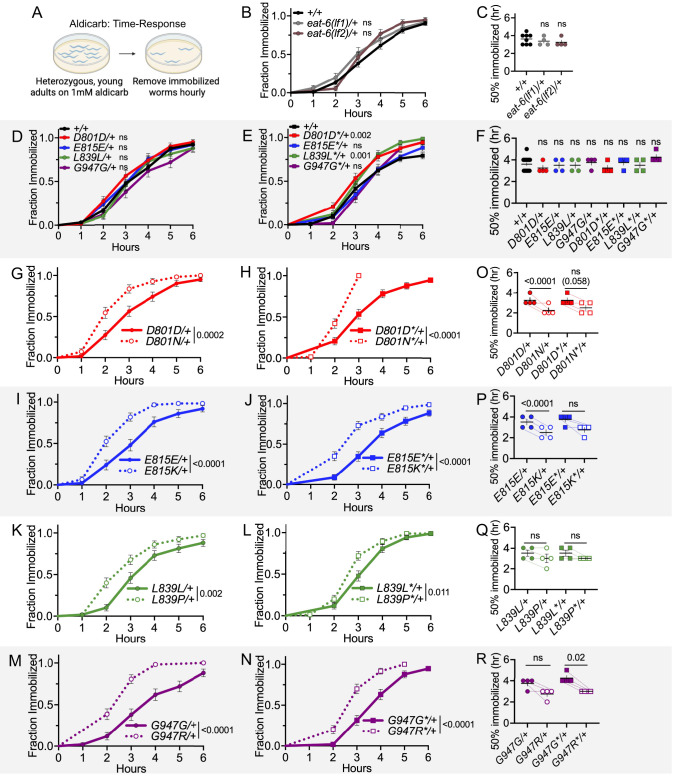
**Dominant NMJ defects of AHC model animals: aldicarb time–response assay.** (A) Schematic of aldicarb time–response assay. The number of heterozygous, young-adult animals immobilized on 1 mM aldicarb was recorded hourly. (B) Immobilization curve for *eat-6(ok1320)/tmC12* (*lf1*/+) and *eat-6(ok1334)/nT1* (*lf2*/+) and balanced wild-type animals. +/+ represents aggregated immobilization curves of *+/nT1* and *+/tmC12* animals. Mantel-Cox log-rank analysis was performed between each balanced *eat-6* allele and corresponding wild-type balanced strain tested in parallel. (C) Median immobilization time for each trial in B. Paired *t*-test between each balanced *eat-6* allele and corresponding wild-type control. (D,E) Immobilization curves for heterozygous CRISPR control primary and replicate strains. +/+ data were aggregated for visual purposes, but Mantel-Cox log-rank analysis was performed on CRISPR control and +/+ animals tested in parallel. (F) Median immobilization time for each trial as shown in D and E. Paired *t*-test between CRISPR control and +/+ strains were run in parallel. (G-N) Immobilization curve for heterozygous primary and replicate CRISPR control and AHC model strains. CRISPR control data are the same as those shown in D and E. Mantel-Cox log-rank analysis between CRISPR control and AHC model strain was tested in parallel. (O-R) Median immobilization time for each trial in G-N. Paired *t*-test between CRISPR control and AHC model strains was run in parallel. For each genotype four biological replicates were tested. For each trial, *n*=10-22 animals per genotype. Error bars indicate mean±s.e.m. ns, not significant (*P*>0.05). All strains shown in D-R have one copy of the *tmC12* balancer, represented by ‘+’.

Different assay formats can reveal subtle differences between genotypes. Therefore, we also assessed the function of the NMJ in an aldicarb time–response format. Here, we measured the cumulative fraction of animals immobilized after exposure to aldicarb (1 mM) every hour, over the course of 6 h (Fig. 4A). Again, we found that heterozygous loss of *eat-6* function did not cause dominant aldicarb hypersensitivity (Figs 4B, 3C; *eat-6(lf1)/+* and *eat-6(lf2)/+* animals; based on Mantel-Cox log-rank test and paired *t*-test of median immobilization time). *eat-6(lf2)* refers to *eat-6(ok1334)*, an in-frame 332 aa deletion (Fig. 1). Heterozygous primary CRISPR control strains were not different from wild-type animals (Fig. 4D). We noticed that replicate D801D*/+ and L839L*/+ animals were hypersensitive to aldicarb in this assay format when compared to balanced wild-type animals, based on a Mantel-Cox log-rank test (Fig. 4E). When median immobilization time is examined, no CRISPR control primary or replicate strain differs from wild type. Background mutations in D801D*/+ and L839L*/+ strains likely have a small effect on NMJ function.

In the time–response assay format, heterozygous animals from all eight primary and replicate AHC model strains still showed a dominant hypersensitivity to aldicarb; they immobilized faster than their CRISPR control strains (based on Mantel-Cox log-rank test, Fig. 4G-N). When assessing these results using paired *t*-tests of median immobilization time, animals from only one of the two independent strains showed dominant aldicarb hypersensitivity for D801N/+, E815K/+ and G947R/+, while neither of the L839P/+ animals showed dominant defects. The Mantel-Cox log-rank analysis may more robustly detect significant aldicarb hypersensitivity as it takes into account the entire immobilization curve, whereas the median immobilization rate is a single parameter. We conclude that inserting AHC patient missense mutations into *eat-6* causes dominant NMJ defects, based on changes to aldicarb sensitivity in heterozygous AHC model animals in two different assay formats. We also conclude that AHC patient mutations do not cause dominant NMJ defects in *C. elegans* due to haploinsufficiency since heterozygous *eat-6(lf)/+* animals always responded normally to aldicarb.

The magnitude of aldicarb hypersensitivity in heterozygous AHC model animals is similar to that of homozygous *eat-6(ad467)* partial loss-of-function animals (Fig. S6). However, since aldicarb hypersensitivity is recessive in homozygous *eat-6* partial loss-of-function animals and dominant in heterozygous AHC model animals, we gained no further insight regarding the nature of AHC mutations solely based on this comparison. The *eat-6* partial loss-of-function mutation and the AHC patient mutations could cause aldicarb hypersensitivity through very different mechanisms.

### Dominant post-synaptic NMJ defects in AHC model animals: levamisole hypersensitivity

**Fig. 5.**
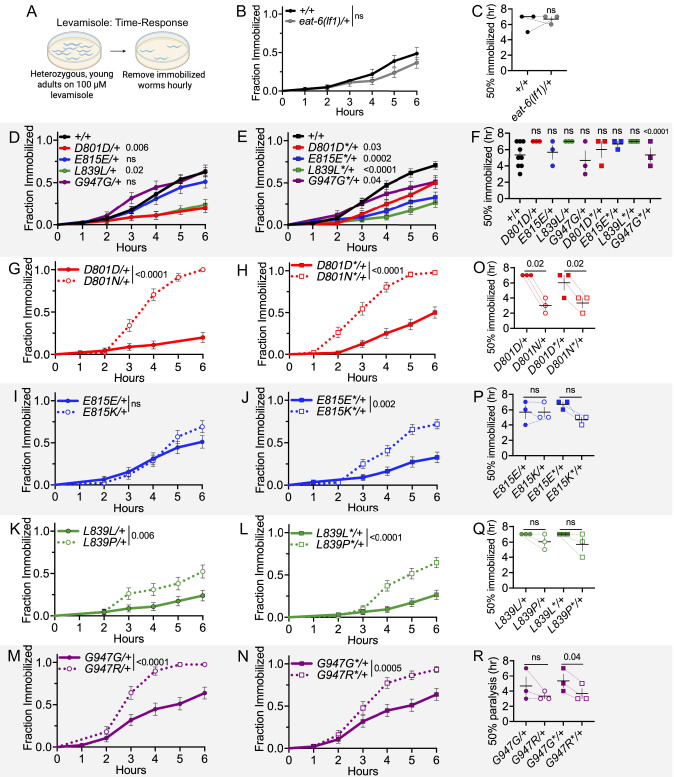
**Dominant post-synaptic NMJ defects of AHC model animals: levamisole time–response.** (A) Schematic of levamisole time–response assay. The number of heterozygous, young adult animals immobilized on 100 dmm levamisole was recorded hourly. (B) Immobilization curve for heterozygous *eat-6(lf1), ok1320* and +/+ animals. Mantel-Cox log-rank analysis. (C) Median immobilization time for each trial as described in B. Paired *t*-test. (D,E) Immobilization curves for +/+ and heterozygous CRISPR control primary and replicate strains. +/+ data were aggregated for visual purposes, but Mantel-Cox log-rank analysis performed on CRISPR control and +/+ animals tested in parallel. (F) Median immobilization time for each trial in D and E. Paired *t*-test between CRISPR control and +/+ strains tested in parallel. (G-N) Immobilization curve for heterozygous primary and replicate CRISPR control and AHC model strains. CRISPR control data are the same as in D,E. Mantel-Cox log-rank analysis between CRISPR control and AHC model strains tested in parallel. (O-R) Median immobilization time for each trial in G-N. Paired *t*-test between CRISPR control and AHC model strains tested in parallel. Data for each genotype are from three biological replicates. For each trial, *n*=9-27 animals per genotype. Error bars indicate mean±s.e.m.; ns, not significant (*P*>0.05). All strains in D-R carry the *tmC12* balancer represented by ‘+’.

Levamisole, an acetylcholine receptor agonist, can reveal post-synaptic NMJ defects in *C. elegans*. Similarly to aldicarb, exposure to levamisole causes immobilization of *C. elegans.* Levamisole mimics acetylcholine to constitutively activate receptors on post-synaptic muscles. In mutant animals, an abnormal rate of immobilization on levamisole indicates defective function of post-synaptic body wall muscles (Lewis et al., 1980a,b). Here, post-synaptic body wall muscle function was assessed using a levamisole time–response assay in which the cumulative fraction of heterozygous animals immobilized on 100 μM levamisole hourly over the course of 5 h (Fig. 5A). Heterozygous *eat-6(lf1)/+* animals responded normally to levamisole (based on Mantel-Cox log-rank test and paired *t*-test of median immobilization time, Fig. 5B,C). When compared to balanced wild-type controls, D801D/+, L839L/+ and all four replicate CRISPR control strains were resistant to levamisole based on a Mantel-Cox log-rank test (Fig. 5D,E). Only G947G*/+ control animals differed from wild type, based on a paired *t*-test of median immobilization times (Fig. 5F). These defects may be due to background mutations and confirms the importance of comparing AHC model animals to their respective CRISPR control strain, especially in a levamisole-sensitivity assay. Heterozygous animals from both primary and replicate heterozygous AHC model strains for D801N/+, L839P/+ and G947R/+ showed hypersensitivity to levamisole, when compared to their CRISPR controls, based on a Mantel-Cox log-rank test (Fig. 5G-N). The replicate heterozygous E815K*/+ strain was also hypersensitive to levamisole when tested this way; only heterozygous E815K/+ animals responded normally to levamisole in the log-rank test (Fig. 5I,J). When median immobilization times were compared in paired *t*-tests, only D801N/+, D801N*/+ and G947R*/+ model animals showed levamisole response defects (Fig. 5O-R). This may indicate that the levamisole response defects in *C. elegans* D801N/+ and, perhaps, G947R/+ AHC model animals are more robust.

Overall, we conclude that all *C. elegans* AHC models show dominant NMJ function defects. For all strains except the primary E815K/+, this includes post-synaptic body-wall-muscle defects. It is important to note that, while levamisole hypersensitivity demonstrates that a post-synaptic muscle defect is present, this does not rule out simultaneous defects in those pre-synaptic neurons that can also contribute to the NMJ defects. Furthermore, we conclude that the dominant post-synaptic muscle defects in heterozygous AHC model animals are caused by a mechanism other than haploinsufficiency, because heterozygous *eat-6(lf1)/+* animals respond normally to levamisole.

### Dominant sleep and arousal defects in *C. elegans* AHC model animals

**Fig. 6.**
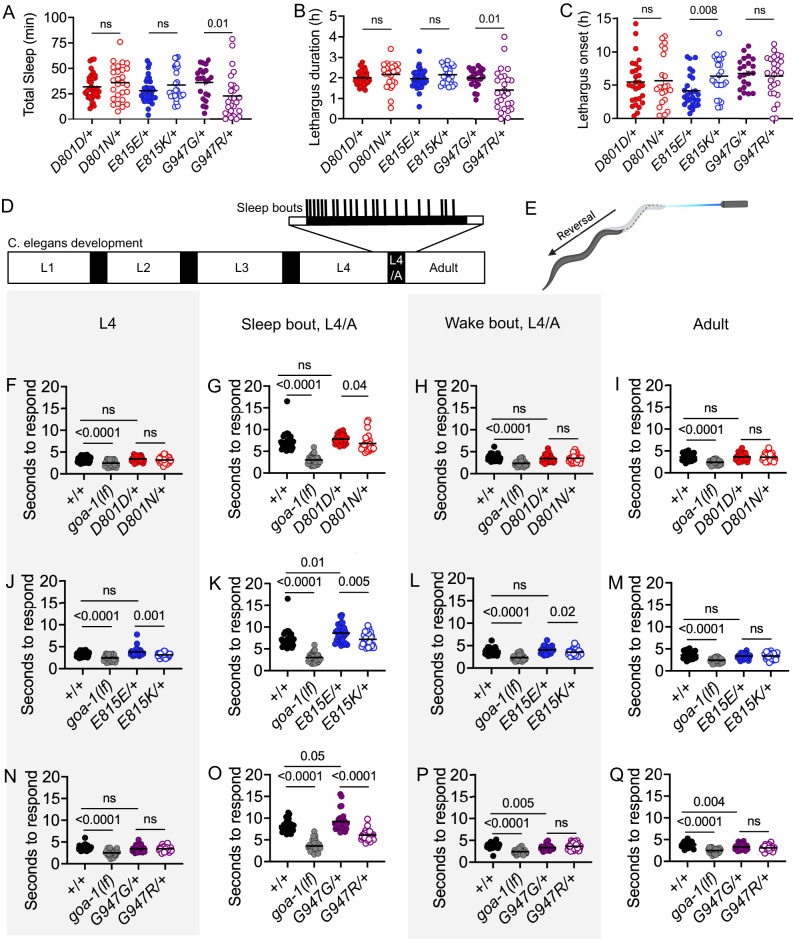
**Dominant sleep and arousal defects in *C. elegans* AHC model animals.** (A) Total sleep time (min), (B) lethargus duration (h), (C) lethargus onset time (h) after recording, recorded in heterozygous CRISPR control and AHC model animals during developmentally timed sleep (DTS) during lethargus at the L4 larval to adult stage transition (L4/A). Welch's unpaired *t*-test between each CRISPR control and AHC model strain. Each AHC model strain was assessed in separate trials, but presented together here for visual purposes. *n*=21-34 animals per genotype. (D) *C. elegans* larval and adult stages (white boxes), separated by lethargus which occurs between stages (black boxes). Results in A-C are from L4/A lethargus. Arousal data in F-Q were collected at the L4, L4/A lethargus, or adult stage. (E) *C. elegans* arousal assay. The time to reverse in response to blue light is recorded. (F-Q) Time to respond to blue light for L4, sleep bout during L4/A, wake bout during L4/A and adult animals. One-way ANOVA with multiple comparison testing with Šidák correction between pairs. *goa-1(sa734lf)* is an internal control for decreased arousal response. *n*=30 animals per genotype, tested in three biological replicates. For D801D/+, D801N/+, E815E/+ and E815K/+ animals, only ‘+’ chromosome is the *nT1* balancer. For G947G/+ and G947R/+ animals, ‘+’ chromosome represents the *tmC12* balancer. Animals indicated by +/+ are the N2 wild-type strain. Error bars indicate mean±s.e.m.

Many AHC patients experience irregular sleeping patterns (Ricci, 1995; Kansagra et al., 2019). Many of the molecular pathways that regulate sleep and arousal are conserved across the animal kingdom, and *C. elegans* exhibit all of the hallmark behavioral changes of sleep during developmentally timed sleep (DTS), which occurs after every larval stage (Trojanowski and Raizen, 2016; Singh et al., 2014). During DTS, animals enter short motionless ‘sleep bouts’ interspersed with ‘waking bouts’ over the course of several hours, in a period called lethargus. Here, we examine sleep in heterozygous AHC model animals during the final lethargus period, as animals transition between L4 and adulthood (hereafter referred to as L4/adult lethargus). We found that, compared to G947G/+ control animals, heterozygous G947R/+ animals spent less total time asleep (Fig. 6A), which can be explained by their shorter lethargus duration (Fig. 6B). Sleep quantity and lethargus duration were normal in heterozygous D801N/+ and E815K/+ model animals compared to control strains (Fig. 6B,C). However, E815K/+ animals entered lethargus 2.2±0.8 h later than E815E/+ control animals (Fig. 6D), which may indicate developmental delay. There was no change in the average sleep bout duration for any of the heterozygous AHC model strains tested (Fig. S4). We conclude that heterozygous G947R/+ animals have dominant defects in sleep duration and that heterozygous E815K/+ animals have delayed sleep onset.

During sleep, arousal thresholds increase, which can be observed by a slower response to mild stimulation, compared to animals that are awake. We measured arousal thresholds in heterozygous AHC model animals at four time points, i.e. L4 larval stage, sleep bout during L4/adult lethargus, wake bout during L4/adult lethargus and adult stage. Arousal thresholds were determined by measuring the time required for a response to an aversive stimulus of blue light (Edwards et al., 2008; Huang et al., 2018). Shorter response times before and after lethargus suggest a global hyperarousal state, whereas shorter response times during lethargus sleep bouts only suggest poor sleep quality. We found that heterozygous D801N/+, E815K/+ and G947R/+ model animals were all easier to rouse during sleep bouts at L4/adult lethargus, compared to their CRISPR controls (Fig. 6G,K,O), suggesting poor sleep quality. At the adult stage, response times for all AHC models did not differ from their controls, suggesting that AHC model animals are not in a global hyperarousal state (Fig. 6I,M,O). In addition to their lethargus sleep bout defects, heterozygous E815K/+ animals also showed arousal defects during L4 larval stage and L4 lethargus wake bouts (Fig. 6J,L). This, together with the delayed lethargus onset observed in E815K/+ animals, suggests that these animals have more difficulty transitioning from wake to sleep state. The dominant arousal defects reported here indicate that sleep is not normal in heterozygous *C. elegans* AHC model animals, even when the quantity of sleep (defined by lack of motion) does not change during lethargus. Each AHC model shows unique defects when sleep and arousal are examined.

### NMJ defects in *C. elegans* AHC model animals that lack NCX-4 function

**Fig. 7.**
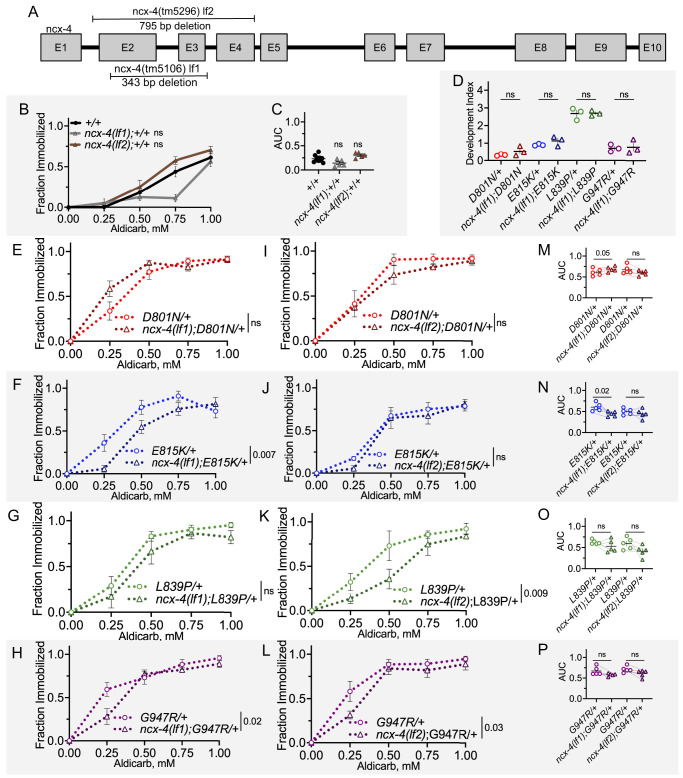
**NMJ defects in *C. elegans* AHC model animals lacking *ncx-4* function.** (A) Schematic of the *ncx-4* gene. *ncx-4(tm5106)* and *ncx-4(tm5296)* are loss of function deletion alleles, here referred to as *lf1* and *lf2*, respectively*.* (B) Aldicarb dose–response curve for *ncx-4*(*lf1);+/+ and ncx-4(lf2);+/+* animals. Each *ncx-4* allele was tested separately and +/+ data from both experiments were aggregated for presentation. Two-way ANOVA analysis performed on *ncx-4* loss of function and +/+ animals tested in parallel. (C) Area under the curve (AUC) values for each trial in B. Paired *t*-test between animals tested in parallel. (D) Development index of homozygous AHC model animals with and without *ncx-4(lf1)*. Welch's unpaired *t*-tests between animals with and without *ncx-4(lf1)* of the same AHC allele. Data were collected from three biological replicates. *n*=114-206 homozygotes per genotype scored. (E-H) Aldicarb dose–response curves for heterozygous AHC model animals with and without homozygous *ncx-4(lf1)*. Two-way ANOVA between genotypes tested together. (I-L) Aldicarb dose–response curves for heterozygous AHC model animals with and without homozygous *ncx-4(lf2)*. Two-way ANOVA between genotypes tested together. (M-P) AUC values from each trial in E-L. Paired *t*-tests between genotypes tested together. For all two-way ANOVA analysis, displayed *P*-value results from genotype as the source of variation. ns indicates *P*>0.05. In all animals, one wild-type ‘+’ chromosome represents the *tmC12* balancer. Data for each genotype were collected from five biological replicates. For each trial, *n*=13-15 animals per genotype were analyzed. Error bars indicate mean±s.e.m.

ATP1A family protein function is deeply conserved across species, and we consider it likely that cellular mechanisms affected in AHC-related defects are similarly conserved. To date, no human genetic modifiers of AHC severity, onset or outcome have been identified, so we turned to modifier genes identified in another animal model. The *Atpα* gene encodes the *Drosophila* ortholog of all four human ATP1A subunits and of *C. elegans eat-6*. In 2014, the Palladino group reported results from a genome-wide *Drosophila* screen that identified genes for which decreased function modified behavioral defects of three different dominant *Atpα* mutations (Talsma et al., 2014). *CJ10*, one of the dominant *Atpα* alleles used in the *Drosophila* screen, serves as the *Drosophila* model for the AHC patient allele G755S, and was first reported in the literature that same year (Ashmore et al., 2009; Sasaki et al., 2014). The Palladino group identified 33 modifier genes, for which decreased function altered the bang-sensitive paralysis defects of heterozygous G755S *Drosophila* AHC model animals (Talsma et al., 2014). Determining which *Drosophila* modifier genes encode cross-species modifiers of ATP1A family proteins should reveal any conserved pathways that are directly relevant to AHC dysfunction. Here, we report cross-species analysis of one *Drosophila* modifier, *Nckx30C*, for which decreased function exacerbated *Drosophila CJ10* dominant bang-sensitive paralysis*. Nckx30C* encodes a transmembrane K^+^-dependent Ca^2+^/Na^+^ exchange protein (NCKX) that is primarily expressed in adult *Drosophila* neurons (Winkfein et al., 2004). Based on aa similarity, the likely *C. elegans* ortholog of *Nckx30C* is *ncx-4*, and the likely human ortholog is *SLC24A2*. We obtained two loss-of-function deletion alleles for *ncx-4*; *ncx-4(tm5106)* and *ncx-4(tm5296)*, referred to here as *ncx-4(lf1)* and *ncx-4(lf2)*, respectively (Fig. 7A).

We first determined whether loss of *ncx-4* function alters the developmental defects in homozygous *C. elegans* AHC model animals. Homozygous AHC model animals also homozygous for *ncx-4(lf1)* showed no alteration regarding developmental defects, and growth defects were not ameliorated or exacerbated (Fig. 7D).

We found that, in heterozygous *C. elegans* AHC model animals, heterozygous loss of *ncx-4* does not alter the dominant aldicarb hypersensitivity of heterozygous D801N/+ or E815K/+ animals (*ncx-4(lf1)/+*, see Fig. S5D-G). For the remainder of the *ncx-4* studies, the impact of homozygous loss of *ncx-4* function was examined. Homozygous loss of *ncx-4* function alone did not alter aldicarb sensitivity, compared to wild-type control animals (two-way ANOVA, Fig. 7B and paired *t*-test of AUC, Fig. 7C).

When we introduced homozygous *ncx-4* loss-of-function alleles into heterozygous AHC models, the effects were small and results varied by allele. Complete loss of *ncx-4* did not affect aldicarb hypersensitivity of D801N/+ animals based on a two-way ANOVA (Fig. 7E,I), but an exacerbation of NMJ defects was detected only in *ncx-4(lf1);D801N/+* animals based on paired *t*-test of AUC values (Fig. 7M). Complete loss of *ncx-4* suppressed aldicarb hypersensitivity in G947R/+ animals; this suppression was observed for both *ncx-4(lf1)* and *ncx-4(lf2)* loss-of-function alleles (two-way ANOVA, Fig. 7H,L). However, when AUC values were assessed in paired *t*-tests, neither *ncx-4(lf1)* nor *ncx-4(lf2)* modified the aldicarb hypersensitivity of G947R/+ animals. In E815K/+ animals, *ncx-4(lf1)* suppressed NMJ defects (two-way ANOVA, Fig. 7F, and AUC paired *t*-test, Fig. 7N), while *ncx-4(lf2)* had no impact. Finally, in L839P/+ animals, only *ncx-4(lf2)* suppressed NMJ defects (two-ANOVA, Fig. 7K, but not AUC pair *t*-test, Fig. 7O), while *ncx-4(lf1)* had no effect. Overall, we noticed that the effect size of *ncx-4* was not large in any case; i.e. if *ncx-4* is a cross-species modifier, it is a weak one. The consistent suppression of NMJ defects in G947R/+ animals – when *ncx-4* function is lost – could reflect unique defects caused by this AHC allele, but the magnitude of the effect is small. Nevertheless, these results demonstrate that dominant NMJ defects in *C. elegans* AHC model animals can be enhanced or suppressed and that there may be conserved genetic mechanisms relevant to AHC defects.

## DISCUSSION

### Development of the first *C. elegans* models of alternating hemiplegia of childhood

We created the first *C. elegans* models of AHC through direct editing of endogenous *eat-6*, which encodes the *C. elegans* ortholog of human ATP1A family proteins. Our models include the three most frequently recurring AHC-associated ATP1A3 mutations in AHC patients (D801N, E815K, G947R) as well as a rare and milder variant (L839P), allowing for comparison of allele-specific phenotypes. Our findings demonstrate that introducing AHC patient missense mutations into *eat-6* causes recessive developmental defects and dominant NMJ defects. Because of abnormal responses to levamisole, post-synaptic muscle defects may contribute to NMJ defects. The developmental, behavioral and NMJ defects reported in this study establish *C. elegans* models of AHC, which can be used as patient genotype archetypes to investigate conserved disease mechanisms, as well as to inform the allele-specific properties and genotype-phenotype relationships of disease-associated ATP1A3 variations.

Current model systems for AHC include HEK293 cell lines, *Xenopus* oocytes, mouse models and patient-derived iPSC neurons (Arystarkhova et al., 2019, 2021; Ng et al., 2021; Uchitel et al., 2021; Li et al., 2015; Simmons et al., 2018). Unfortunately, none of the current *in vitro* assays correlate with allele-specific disease severity (Lazarov et al., 2020), and generating mouse models for the numerous AHC-associated mutations in ATP1A3 is laborious and expensive. The *C. elegans* AHC models are complementary to the current AHC model systems and can immediately be used for compound screening, identification of genetic suppressors or testing hypotheses regarding allele-specific suitability of different therapeutic approaches.

When these studies are undertaken, best practice will be to compare results in heterozygous *C. elegans* AHC models to their heterozygous CRISPR controls, rather than using balanced wild-type animals as controls. This is especially important in levamisole-sensitivity assays, as multiple CRISPR control strains were resistant to levamisole when compared to wild type (Fig. 5D,E). As the use of CRISPR control strains may not be convenient in all assay formats (e.g. large-scale screening), we re-analyzed the aldicarb and levamisole sensitivity data as shown in Figs 3, 4 and 5, by comparing balanced heterozygous AHC animals to balanced wild-type ‘+/+’ animals that were tested in parallel (Dataset 1, ‘Alt. Analysis Figs. 3-5’). For aldicarb assays, interpretations of data do not change; all primary and replicate heterozygous AHC model strains still show robust dominant aldicarb hypersensitivity when analyzed in a two-way ANOVA or log-rank test. For levamisole sensitivity assays, the post-synaptic defects observed in E815K/+ and L839P/+ animals are no longer significant, when compared to balanced wild-type +/+ animals as a control in a log-rank test. Combined, these results suggest that background mutations may have been introduced during CRISPR editing or that the ‘silent edits’ in CRISPR control strains impact phenotypes in some assays, because we sometimes detected differences between balanced CRISPR control and balanced wild-type animals (Figs 4E, 5D,E, 6K,O,P,Q).

### Dominant NMJ defects in *C. elegans* AHC models are inconsistent with haploinsufficiency

It is well established that dominant AHC-causing variants in ATP1A3 decrease Na^+^/K^+^-ATPase activity (Li et al., 2015; Simmons et al., 2018; Immanneni et al., 2024). However, multiple lines of evidence suggest that AHC mutations cause additional antagonistic effects that contribute to AHC pathophysiology. D801N, E815K and G947R mutations in ATP1A3 have dominant-negative effects on activity of wild-type ATP1A3 in *Xenopus* oocytes (Li et al., 2015). In HEK293 cell lines, expression of AHC-associated ATP1A3 variants cause immature ATP1B1 beta subunits to accumulate in the endoplasmic reticulum and/or Golgi (Sweadner et al., 2019; Arystarkhova and Sweadner, 2024; Arystarkhova et al., 2021). In *C. elegans*, EAT-6 also plays a role in the expression and localization of post-synaptic receptors in a manner that is independent from ATPase activity (Doi and Iwasaki, 2008), which could help explain NMJ defects observed in AHC model animals. Last, the behavioral defects in mice heterozygous for ATP1A3 knockout are not as severe as the defects of heterozygous AHC model mice, suggesting a mechanism beyond simple loss of ATPase function contributes to defects (Sugimoto et al., 2018; Terrey et al., 2024 preprint).

The *C. elegans* AHC models described here provide additional support for the hypothesis that AHC patient mutations disrupt cellular function beyond haploinsufficiency. Here, all eight heterozygous AHC model primary and replicate strain animals showed robust dominant NMJ defects in both aldicarb time–response and dose–response assays (Figs 3 and 4), as well as in levamisole time–response assays (except for E815K/+, Fig. 5). This is in stark contrast to heterozygous *eat-6(lf)/+* animals, which always showed normal sensitivity to aldicarb and levamisole (Figs 3B,C, 4B,C, 5B,C). Given that heterozygous *eat-6(lf)/+* animals did not exhibit dominant NMJ defects, but all heterozygous AHC model animals did, we conclude that NMJ function in heterozygous AHC model animals must be disrupted through a mechanism beyond simple loss of pump function. Thus, we rule out haploinsufficiency as an explanation for NMJ defects in heterozygous AHC model animals.

Because heterozygous AHC model *C. elegans* have dominant NMJ defects that are not solely due to decreased pump function, the AHC mutations are classified as genetic ‘gain-of-function’ alleles. By definition, genetic gain-of-function alleles can be hypermorphic (increased EAT-6 activity), antimorphic (dominant-negative, antagonizing normal EAT-6 function), and/or neomorphic (novel activity, unrelated to normal EAT-6 function). Because homozygous *eat-6* decreased function alleles are hypersensitive to aldicarb (Doi and Iwasaki, 2008; Sorkaç et al., 2016; Govorunova et al., 2010), a hypermorphic allele would be expected to cause aldicarb resistance – but heterozygous AHC model animals are hypersensitive to aldicarb (Figs 3, 4). Thus, increased EAT-6 activity is an unlikely explanation for dominant NMJ defects in heterozygous AHC model animals. Results presented here cannot distinguish between gain-of-function antimorphic and/or neomorphic actions caused by AHC variants. Either of these could potentially explain the observed dominant NMJ defects that go beyond haploinsufficiency or reduced EAT-6 function.

We see further evidence that AHC patient mutations can cause defects beyond loss of pump activity when considering the developmental defects of homozygous AHC model animals. Homozygous *eat-6(lf1)* animals have severe development defects that are similar to those observed in homozygous E815K animals. However, development defects in homozygous D801N and G947R animals were more severe than those in homozygous *eat-6(lf1)* animals (Fig. 2C,D). For D801N and G947R animals, this is further evidence that a mechanism beyond simple loss of ATPase activity is likely contributing to homozygous development defects.

### *C. elegans* AHC models have allele-specific impairments

All AHC alleles likely share a common disease mechanism but there may be additional allele-specific defects. In AHC patients, there is a wide spectrum of symptom presentation and severity (Panagiotakaki et al., 2015; Viollet et al., 2015; Yang et al., 2014; Vezyroglou et al., 2022); the *C. elegans* AHC models also differ in their defects. Most notably, L839P is a comparatively weaker allele in patients (Pratt et al., 2020; Yang et al., 2014), and homozygous L839P *C. elegans* AHC model animals had the least-severe survival and development defects (Fig. 2B-D). Despite exhibiting no growth past the L1 stage, homozygous L839P animals survived for at least 10 days while the vast majority of homozygous D801N, E815K and G947R larvae died within 24 h. For reference, under these conditions wild-type *C. elegans* reach the mid-L1 stage by 20 h and reach adulthood within 3 h. Because the eight primary and replicate heterozygous AHC model strains were not examined in the same trials on the same days, it is difficult to directly compare the severity of dominant aldicarb response defects between the AHC models. However, dominant NMJ defects in primary and replicate L839P/+ model animals may be weaker (according to aldicarb time–response; Fig. 4K,L,Q).

While at least one strain from all four AHC model animals showed a dominant hypersensitivity to levamisole, indicating post-synaptic body-wall muscle defects, the defect in heterozygous D801N/+ and maybe G947R/+ animals were arguably more severe. A levamisole-response defect could only be detected in a paired *t*-test of median immobilization time for D801N/+, D801N*/+ and G947R*/+ model animals (Fig. 5). In humans, cardiac muscle impairment is observed much more frequently in patients carrying the D801N allele; patients carrying the D801N variant often have short QT syndrome and have an increased risk of life-threatening arrhythmias and sudden cardiac death (Moya-Mendez et al., 2021). *C. elegans* do not have a heart but their body-wall muscle shares key contractile properties with vertebrate cardiac muscle. It is interesting that the AHC patient mutation that most frequently causes cardiac muscle defects in patients is also the *C. elegans* model with the strongest and most reproducible post-synaptic muscle defect.

Allele-specific defects in the *C. elegans* AHC models are also apparent when sleep and arousal are examined. All *C. elegans* AHC model animals have decreased arousal thresholds during lethargus sleep bouts, which suggests poor sleep quality, altered sensory circuit function or an imbalance of neuromodulators (Cho and Sternberg, 2014; Scheer and Bargmann, 2023). However, only heterozygous G947R/+ animals showed an overall decrease in sleep quantity (Fig. 6). Additionally, only heterozygous E815K/+ model animals had a shorter L4/adult lethargus duration, decreased arousal thresholds in L4 and decreased wake bouts during lethargus, which suggests difficulties transitioning between wake and sleep states or may indicate broader developmental defects.

Many factors could explain how different alleles in the same protein can lead to varying phenotypes and severities. The physical location of each point mutation within the Na^+^/K^+^ ATPase may affect the extent to which pump function is decreased. For example, D801N and G947R mutations are located in transmembrane domains near ion binding sites, while E815K and L839P mutations are located further away in intracellular loops (Li et al., 2015). Furthermore, alleles may induce different antagonistic pathways that depend on the extent of ion gradient disruption, cascading effects in the ER/Golgi (Arystarkhova et al., 2019, 2021; Arystarkhova and Sweadner, 2024) or the role of the Na^+^/K^+^ ATPase in localization of post-synaptic receptors (Doi and Iwasaki, 2008). We must consider the possibility that each AHC patient allele responds differently to genetic modifiers or chemical intervention due to allele-specific impairments.

### Loss of *ncx-4* function inconsistently modifies NMJ defects across alleles in *C. elegans* AHC models

The conservation of neurological genes and pathways as well as the ease of genetic manipulation in *C. elegans* mean that these AHC models may be a powerful tool for investigating AHC pathophysiology. Foundational work by the Palladino lab in 2014 identified 33 genetic modifiers of a *Drosophila* G755S model of AHC (Talsma et al., 2014). We found cross-species modification of AHC-related phenotypes for some AHC alleles when the function of a conserved NCKX was lost. *ncx-4* encodes a K^+^-dependent Ca^2+^/Na^+^ exchanger that is primarily expressed in adult neurons (Winkfein et al., 2004). Two different loss-of-function alleles in *ncx-4* suppressed dominant aldicarb dose–response defects in heterozygous G947R/+ animals. However, only one of the two *ncx-4* loss-of-function alleles suppressed NMJ defects in heterozygous E815K/+ and L839P/+ animals. In all cases, the extent of suppression by *ncx-4* loss-of-function alleles was small. It is possible that *ncx-4* is a weak suppressor for all three AHC alleles, making it difficult to reproducibly detect subtle changes in aldicarb sensitivity. Alternatively, due to allele-specific impairments, loss of *ncx-4* function might only reliably modify defects in G947R/+ animals.

In the *Drosophila* AHC model, heterozygous loss of *Nckx30C* function enhanced bang-sensitive paralysis defects, while homozygous loss of *ncx-4* function sometimes suppressed NMJ defects in the *C. elegans* AHC models. These are very different behaviors; *Drosophila* bang-sensitive paralysis behavior is regulated through different circuitry and pathways than *C. elegans* NMJ function. When assessing different behaviors in multiple species, the perturbation of a conserved gene could exacerbate one behavior and ameliorate another. Overall, if *ncx-4* is a cross-species modifier, we conclude that calcium homeostasis could play a role in AHC pathophysiology and that *C. elegans* AHC models can be used to identify conserved genetic mechanisms relevant to AHC. We plan to implement this same approach to test the remaining candidate modifier genres from the *Drosophila* screen in the *C. elegans* AHC models.

## MATERIALS AND METHODS

### *C. elegans* strains and maintenance

All strains were reared at 20°C on standard Nematode Growth Medium (NGM) seeded with OP50 *E. coli*, unless otherwise indicated. All assays used age-matched hermaphrodites. See Dataset 1, ‘Strain list’. Heterozygous AHC model and control strains were balanced by either the *tmC12[myo-2p::mCherry]* (Dejima et al., 2018) or *nT1[qIs51 (myo-2p::GFP, pes-10p::GFP, F22B7.9p::GFP)]* (Ferguson and Horvitz, 1985; Clark et al., 1988; Edgley et al., 2006) balancer chromosomes. The *nT1* balancer became unreliable in progeny produced by older hermaphrodites and many balanced progenies lacked GFP expression. Thus, *tmC12* balanced strains were used unless otherwise stated in the figure legend. Heterozygous AHC model animals balanced over *nT1* or *tmC12* grew from egg to L4 roughly 12 h slower than CRISPR control animals.

### Statistical analysis

All experiments were performed by observers unaware of the genotype and, when possible, treatment. Quantitative data were analyzed using GraphPad Prism 10. In figures, *P*-values <0.05 are displayed and ns indicates *P*>0.05. Unrounded *P*-values can be found in Dataset 1. All independent trials are biological replicates conducted on different days, using different animals. Even when data are aggregated in figure panels for visual purposes, statistical comparisons were only made between strains tested in parallel on the same day.

Usually, balanced AHC model, CRISPR control, wild-type and *eat-6(lf)* animals were tested together in aldicarb and levamisole assays. When figure panels were assembled, we separated results for loss-of function alleles, CRISPR controls and AHC alleles. Therefore, sometimes the same balanced wild-type and CRISPR control animals are shown in multiple panels. Each trial has a unique number in Dataset 1. For aldicarb and levamisole assays, we use two-tailed paired *t*-tests to control for day-to-day variation in culture conditions, environmental factors and assay-plate preparation.

### CRISPR-Cas9 microinjection to generate AHC model and CRISPR control strains

Basic Local Alignment Search Tool (BLAST) was used for amino acid alignment of human ATP1A3 (NP_689509.1) and *C. elegans eat-6* (B0365.3), as shown in Fig. S1. AHC model (D801N, E815K, L839P, G947R) and control (D801D, E815E, L839L, G947G) strains were generated using CRISPR-Cas9 homology-directed repair of endogenous *eat-6* with a single-stranded oligonucleotide template by using previously published methods (Dokshin et al., 2018; Ghanta et al., 2021; Kim et al., 2014). Two Alt-R CRISPR-Cas9 sgRNA (Integrated DNA Technologies) were used to target either side of each edited region in *eat-6*. Repair templates were Alt-R homology-directed repair (HDR) donor single-stranded oligodeoxynucleotides (ssODNs) (Integrated DNA Technologies), containing the AHC missense mutation, silent edits to generate restriction sites, silent edits to destroy PAM sites and up to 30 bases of unedited, flanking homology arms (Fig. S2 and Dataset 1). Editing was confirmed using both PCR genotyping and Sanger sequencing (Eurofins Genomics). Primary and replicate AHC model and CRISPR control strains (derived from different F1 animals post-CRISPR editing) were arbitrarily selected then backcrossed to wild-type animals at least twice before analysis.

### PCR genotyping

All primers were ordered from Integrated DNA Technologies. One*Taq* DNA Polymerase was used in all PCRs. Dataset 1 contains a full list of primers used to genotype AHC model and CRISPR control strains.

### Homozygous development and growth assessment

Ten to twenty gravid adult *C. elegans* were picked, transferred onto three freshly seeded NGM plates and allowed to lay eggs at 20°C for 3 h; the total number of eggs per genotype was counted. After 18 h at 20°C, homozygous animals were categorized as: unhatched, dead L1, live L1, motile L1 or motile L1 expressing *lin-4p::GFP*. Live L1 animals had spontaneous nose or tail twitches or minimally twitched in response to gentle prodding with a platinum wire. Motile L1 animals showed muscle movement throughout their entire body and were able to crawl forward any distance either spontaneously or after gentle prodding. Dead L1 animals had hatched but did not respond to prodding. *myo-2p::mCherry* fluorescence from the *tmC12* balancer ‘+’ could not be detected in the egg, so any unhatched eggs after 18 h were assumed to be homozygous unbalanced animals. For the development index, each of the five categories was given a category score between 0 and 5, from most severe (0) to least severe (5). The numbers of homozygous animals in each category multiplied by the category score were added up and divided by the total number of homozygote animals to produce a development index score for each trial. For each genotype, between 117 and 211 homozygous animals were scored across three biological replicates. Data were analyzed using one-way ANOVA with Tukey's multiple comparisons test between all possible pairs. At 18 h after egg laying, some homozygous L1 animals were immobilized with 2,3-butanedione monoxime (BDM) (30 mg/ml in M9 buffer) and mounted on 2% agarose pads. Images were captured under a 40× objective on a Zeiss AxioImager ApoTome, using AxioVision software v4.8.

### Pharyngeal pumping

*C. elegans* were picked at L4 stage and assayed as adults after 1 day or 8 days. Videos of individual animals were recorded for 10 s on a PointGrey camera using FlyCapture 2. Full or partial grinder movement in any direction was manually counted as a pharyngeal pump. The number of pumps per 10 s video was counted and converted into pumps/minute. Between 26 and 30 animals in total were recorded across three biological replicates. Data were analyzed using one-way ANOVA with multiple comparison testing with Šidák correction between each AHC model and its respective CRISPR control strain.

### Egg laying

Eight age-matched day-1 adult *C. elegans* laid eggs on freshly seeded NGM plates for 6 h at 20°C and the total number of eggs was counted. Data were collected from three biological replicates and analyzed using one-way ANOVA between +/+ and two replicate AHC model strains, and their two replicate CRISPR control strains. Multiple comparison testing with Šidák correction was used between each +/+ and CRISPR control strain, and between each AHC mutant and its respective CRISPR control. Additionally, paired *t*-test was performed to determine the statistical significance of differences between N2 and *eat-6(ad467)* animals, and between *+/+* and *eat-6(lf2)/+* animals.

### Growth on *E. coli* strains DA837 or HB101

Bacterial lawns of *E. coli* strains DA837, HB101 or OP50 were seeded onto NGM plates and left to dry overnight before introduction of age-synchronized L1 *C. elegans* from a bleach prep (Porta-de-la-Riva et al., 2012). The growth rates, locomotion and overt behavior of heterozygous and homozygous AHC model animals were then qualitatively compared.

### Long-term temperature stress

*C. elegans* were reared at 12°C or 27°C for at least three generations and observed for overt locomotion defects or changes to survival, growth, and development.

### Acute heat shock

Twenty *C. elegans* per genotype were heat shocked at 35°C for 2.5 h on seeded NGM plates placed agar-side up on a heat block and then moved to 20°C for recovery. 57 to 64 h later, survival was scored based on pharyngeal pumping and spontaneous or evoked motion. Data were collected from three biological replicates with 18-20 animals per genotype per trial. One-way ANOVA with multiple comparison testing with Šidák correction was used to determine the statistical significance of differences between CRISPR controls and AHC model animals.

### Acute cold shock

Twenty animals per genotype were cold shocked at either 4°C for 17 h or 0°C for 4 h. For the 4°C cold shock, seeded NGM plates were placed in the refrigerator in a singular layer. For the 0°C cold shock, plates were individually sealed in parafilm and buried in ice in a single layer. After either cold shock, plates were incubated at 20°C for 18 h. After 18 h, survival was scored on the basis of pharyngeal pumping and spontaneous or evoked locomotion. Data were collected from three biological replicates, each with 20 animals per genotype.

### Aldicarb dose–response assay

The aldicarb dose–response assay was adapted from previous work (Nonet et al., 1997). One day prior to the assay, L4 hermaphrodites were selected and allowed to grow overnight on normal seeded NGM plates. NGM plates containing 0, 0.25, 0.5, 0.75, and 1 mM of aldicarb were prepared and left to dry on the benchtop overnight. On the day of the assay, a copper ring was melted onto the center of each aldicarb plate, and 10 μl of *E. coli* strain OP50 was seeded in the center of the ring (Hawkins et al., 2015). Once the *E. coli* OP50 strain had dried, 10-15 day-1 adult *C. elegans* of each genotype were transferred onto aldicarb plates of each dose. The plates were left for 5 h at room temperature on the benchtop. After 5 h, immobilization was scored by gently tapping animals twice on the head and twice on the tail with a platinum wire. If animals did not move, twitch or pump after 3 s of observation, they were scored as immobilized. Escaping animals were censored. After five biological replicates, immobilization curves were analyzed using two-way ANOVA. Area under the curve (AUC) values were calculated, and mean differences were analyzed in paired *t*-tests. Only strains tested in parallel on the same days were compared.

### Aldicarb time–response assay

The aldicarb time–response assay was adapted from previous work and largely undertaken as described above for the dose–response assay. However, only 1 mM aldicarb (Mahoney et al., 2006) NGM plates was used and immobilization was assessed every hour for 6 h. Four biological replicates of 10-22 *C. elegans* per genotype per replicate were analyzed using a Mantel-Cox log-rank test for two strains tested in parallel. The hour when at least 50% of animals were first immobilized was recorded, and mean differences were analyzed in paired *t*-tests for two strains assayed in parallel. If a strain did not reach 50% immobilization by the final 6-h timepoint, the 50% immobilization time was arbitrarily set as 7 h. Only strains tested in parallel on the same days were compared.

### Levamisole time–response assay

The levamisole time–response assay was adapted from previously described methods (Gottschalk et al., 2005; Hawkins et al., 2015; Doi and Iwasaki, 2008) to be conducted similarly to the aldicarb time–response assay described above. Immobilization was examined on 100 µM levamisole NGM plates every hour for 6 h. Three biological replicates of 9 to 27 *C. elegans* per genotype per replicate were tested, and mean differences in survival were analyzed using a Mantel-Cox log-rank test performed between two strains tested in parallel.

### Developmentally timed sleep

*C. elegans* were visually staged at L4.2 (Mok et al., 2015) and imaged for 12 h, including L4/A lethargus in individual polydimethylsiloxane (microfluidic chips containing ten chambers, as described previously by Huang et al., 2017) chambers. Images were taken every 10 s, and image subtraction was used to detect lack of motion as previously described (Huang et al., 2017). Up to four genotypes were tested per imaging session, and controls were pooled with other strains tested within the same 24-h period. Each genotype was run in at least three sessions. In total, 22 to 34 animals were examined per genotype. Mean differences between AHC model animals and appropriate CRISPR controls were analyzed using paired *t*-tests.

### Arousal

With modifications, arousal threshold assays were performed as previously described (Huang et al., 2017). Five to ten gravid adult *C. elegans* were transferred to a freshly seeded NGM plate and placed at 20°C for 48 h. Plates were then left under a dissection microscope light for 30 min to acclimate animals to the temperature and light. Plates were viewed at 4× magnification by using a dissection microscope with the lid on. A 5 mW, a 405 nm laser affixed with a ∼0.5 mm pinhole cover was mounted to a magnetic manipulandum arm and positioned to emit light to the center of the microscope field of view. The tip of the laser pointer was fixed 3 cm above the lid of the plate as vertically as the scope permitted. Current was maintained at 0.28 A to ensure a consistent laser intensity of ∼100 lux. Sleeping and awake animals were positioned with their pharynx in the center of the field of view, and the laser was activated until animals responded by moving one body bend forwards or backwards. In moving animals, a change in direction or an acceleration by one body bend was considered a response. Laser illumination was terminated when a response was detected, and response latency times and direction of response (forward or reverse) were digitally recorded. Animals were visually staged as L4.2 (L4), L4.6-L4.8 (sleep bout L4/A), L4.6-L4.8 (wake bout L4/A), and vulva-everted young-adults (adult). Sleep was defined as absence of moving and feeding behavior. In total, thirty animals of each genotype at each stage were assayed in three biological replicates. Data were analyzed using a one-way ANOVA with multiple comparison testing with Šidák correction, comparing data from +/+ and *goa-1(lf)*, +/+ and CRISPR control, or CRISPR control and AHC model strain. A separate ANOVA test was used for each AHC model strain and its corresponding controls that were assayed together.

## Supplementary Material

10.1242/dmm.052809_sup1 Supplementary information

Dataset 1.
